# Mothers’ experiences of support during a structured breastfeeding support programme in antenatal care: a lifeworld hermeneutic study

**DOI:** 10.1186/s13006-026-00817-w

**Published:** 2026-02-05

**Authors:** Ingrid Blixt, Ove Axelsson, Karin Enskär, Eva-Lotta Funkquist

**Affiliations:** 1 Department of Women’s and Children’s Health, Uppsala University, Dag Hammarskjölds väg 14 B, Uppsala, 752 37 Sweden; 2 Centre for Clinical Research Sörmland, Uppsala University, Eskilstuna, Sweden

**Keywords:** Antenatal care, Baby-Friendly hospital initiative, Breastfeeding, Expectant mothers, Experiences, Intervention, Mothers, Support, Qualitative methods

## Abstract

**Background:**

Step three of the Ten Steps to Successful Breastfeeding encourages midwives in antenatal care to discuss the importance and management of breastfeeding with expectant and new parents. However, there is a gap in the literature regarding mothers’ experiences of individual antenatal breastfeeding education. This study aims to explain and understand the meaning of the research phenomenon: support from midwives and partners during a structured breastfeeding support programme in antenatal care. The lived experiences of this phenomenon are interpreted from the perspectives of expectant mothers (during pregnancy) and new mothers (two months postpartum).

**Methods:**

The analysis employed a lifeworld hermeneutic approach, based on diaries and interviews with 20 mothers conducted during pregnancy and two months after birth. Participants received structured breastfeeding support based on the Ten Steps to Successful Breastfeeding. A purposive sample was recruited in 2021 in Sweden.

**Results:**

The research phenomenon, support from midwives and partners during a structured breastfeeding support programme in antenatal care, is understood through the following themes: Feeling safe through responsive, individualised dialogue; Grounding confidence through tailored and accessible breastfeeding knowledge; Breastfeeding as an existential anchor for emotional closeness and maternal identity; Co‑creating space for breastfeeding, with partner support as negotiated meaning; Weighing breastfeeding as a lived balance of perceived gains and everyday constraints; Trust as a foundation for perseverance and acceptance; and Feeling left behind when dialogue and continuity are missing. The main interpretation can be understood as being prepared to make independent decisions about breastfeeding.

**Conclusions:**

Being prepared to make independent decisions about breastfeeding extends beyond receiving information and is shaped through responsive, meaningful encounters. Individual dialogue grounded in each mother’s lived experiences, needs, and circumstances fostered a sense of safety, reflection, and growing confidence throughout pregnancy and early motherhood. Mothers valued support that recognised their perspectives and strengthened their ownership of breastfeeding decisions, while negotiated partner involvement deepened shared understanding without compromising maternal autonomy. The findings highlight that continuity‑based, individual antenatal breastfeeding education can complement group sessions, enhance maternal satisfaction, and enable midwives to provide more equitable and accessible care for all families.

**Trial registration:**

ACTRN12623000648628. Date registered 15th June 2023. Retrospectively registered.

**Supplementary Information:**

The online version contains supplementary material available at 10.1186/s13006-026-00817-w.

## Background

The United Nations Convention on the Rights of the Child (UNICEF) emphasises that all infants have the right to the highest attainable standard of health [1]. Breastfeeding provides both short- and long-term health benefits for mothers and infants and is the best form of nutrition unless health issues prevent it. It protects infants against lower respiratory tract infections, severe diarrhoea, otitis media, childhood obesity, childhood leukaemia, type 2 diabetes, and overweight, and also contributes to increased IQ [2]. Consequently, the World Health Organization (WHO) and UNICEF recommend that breastfeeding be initiated within one hour of birth, with exclusive breastfeeding for the first six months [3].

In Sweden, approximately 100,000 infants are born each year [4], and the great majority (93%) initiate breastfeeding shortly after birth [5]. However, exclusive breastfeeding rates have declined over time, and the mother’s level of education influences whether she exclusively breastfeeds her infant. For instance, 73% of Swedish mothers with a university education exclusively breastfeed one month after birth, compared with 61% of mothers with a high school education [4].

The WHO and UNICEF launched the Baby-friendly Hospital Initiative (BFHI) in 1991 to support healthcare services worldwide in implementing the Ten Steps to Successful Breastfeeding (Ten Steps) (Table 1) [6]. These Ten Steps emphasise that parents need access to evidence-based information and support from the healthcare system, as well as protection from commercial interests that may negatively impact breastfeeding [6]. A systematic review has demonstrated a dose-response relationship between the number of BFHI steps implemented and continued breastfeeding during the first months after birth [7].

**Table 1:** Ten steps to successful breastfeeding

**Critical management procedures**
**1a.** Fully comply with the *International Code of Marketing of Breast-milk Substitutes* and relevant *World Health Assembly* resolutions
**1b.** Have a written infant feeding policy that is routinely communicated to staff and parents
**1c.** Establish ongoing monitoring and data management systems
**2.** Ensure that staff have sufficient knowledge, competence and skills to support breastfeeding
**Key clinical practices**
**3.** Discuss the importance and management of breastfeeding with expectant mothers and their families
**4.** Facilitate immediate and uninterrupted skin-to-skin contact, and support mothers to initiate breastfeeding as soon as possible after birth
**5.** Support mothers to initiate and maintain breastfeeding and to manage common difficulties
**6.** Do not provide breastfed newborns with any food or fluids other than breast milk, unless medically indicated
**7.** Enable mothers and their infants to remain together and to practise rooming-in 24 h a day
**8.** Support mothers to recognise and respond to their infants’ cues for feeding
**9.** Counsel mothers on the use and risks of feeding bottles, teats and pacifiers
**10.** Coordinate discharge so that parents and their infants have timely access to ongoing support and care

Step three of the Ten Steps encourages midwives in antenatal care to discuss the importance and management of breastfeeding with expectant parents [6]. A systematic review of the effectiveness of antenatal breastfeeding education concludes that it increases mothers’ knowledge of breastfeeding. Mothers with positive attitudes and greater knowledge are more likely to initiate and continue breastfeeding. The review also emphasises the need for further research to determine whether group or individual antenatal breastfeeding education is more effective [8].

Quality of care refers to the extent to which the healthcare system increases an individual’s likelihood of achieving desired health outcomes. Information and support should be evidence-based; adapted to individuals’ preferences, needs, and values; and consistent in quality, regardless of ethnicity or socio-economic status [9]. In Sweden, antenatal educational programmes are part of midwives’ mission in antenatal care [10] and are delivered in various formats: face-to-face or digital large-group sessions, online or face-to-face small-group sessions, or individual face-to-face sessions [4].

In 2023, the National Board of Health and Welfare in Sweden recommended antenatal educational programmes in small-group sessions [11]. These programmes should cover childbirth, the immediate period after childbirth, the mother’s physical and mental health, couple relationships, parenting, and breastfeeding. The Board also recommends that midwives provide individual breastfeeding sessions for expectant mothers, based on the Ten Steps [11]. However, a mixed-methods systematic review found that mothers were not always satisfied with the education and support they received [12].

In Sweden, antenatal education in small groups is mainly offered to first-time parents, with participation decreasing over recent years. During the COVID-19 pandemic, participation dropped further and has not yet recovered. In 2023, fewer than 17.7% of first-time mothers participated in small-group antenatal educational programmes [4]. Young, single, or less educated mothers tend to find the programmes less helpful and participate to a lesser extent [13]. Previous research on mothers’ experiences of antenatal education has primarily focused on group sessions and shows that these often focus on childbirth [14–16]. A Swedish study showed that first-time mothers had few questions and were unsure what breastfeeding knowledge they would need after birth [14]. Studies from Malaysia and Switzerland found that mothers were dissatisfied because the breastfeeding education was too brief and often idealised by the professionals [15, 16].

There is a gap in the literature regarding expectant and new mothers’ experiences of individual antenatal breastfeeding education. A better understanding of expectant and new mothers’ perspectives on support during individual face-to-face visits in antenatal care, based on the Ten Steps, could help healthcare professionals improve the support they offer to parents. This study aims to explain and understand the meaning of the research phenomenon: support from midwives and partners during a structured breastfeeding support programme in antenatal care.

## Methods

### Design

An exploratory, longitudinal, and qualitative design was chosen, using diary entries and semi-structured individual interviews conducted during pregnancy and two months after the infant’s birth. A lifeworld hermeneutic approach, as described by Dahlberg et al. [17], was employed to explore and understand the meaning of support during a structured breastfeeding support programme. The lived experiences of this phenomenon are interpreted from the perspective of expectant mothers (during pregnancy) and new mothers (two months postpartum). The study includes mothers who participated in The Breastfeeding Study [18, 19], in which couples were offered structured breastfeeding support based on the Ten Steps [6]. Data were collected between March and December 2021.

The lifeworld hermeneutic approach builds on the ideas of the philosophers Husserl, Heidegger, Gadamer, and Ricoeur regarding phenomenology and hermeneutics [17, 20–24]. This approach requires researchers to adopt an open attitude towards the phenomenon and to set aside their assumptions and prior knowledge throughout the research process. Gadamer [20] asserts that researchers should strive for openness by attempting to set aside their pre-understandings in order to comprehend something new and different in their research. According to Ricoeur [21], the process of interpretation includes distancing, questioning, and adopting a critical approach. The process involves suggestions on how to explain and understand meanings.

### Intervention

The study setting and the regional healthcare system have previously been described in detail [18]. The study was conducted in a central region of Sweden, where the healthcare organisation provides care for approximately 3,300 infants per year. This region faces significant socio-economic challenges. A considerable proportion of mothers have a low level of education (59%), are aged 25 years or younger (13%), and a high proportion are foreign-born (35%). Most mothers in the region (69%) exclusively breastfeed for at least one week.

Midwives working at antenatal clinics in the intervention group participated in a breastfeeding training programme aligned with the Ten Steps to Successful Breastfeeding and WHO recommendations on breastfeeding [18]. The training included guidance on using the pictures and conversation materials designed to promote empathetic, reflective listening through open-ended questions, reflection, and summarisation. Midwives were encouraged to explore what the expectant mother already knew about breastfeeding, obtain consent before providing information, and offer tailored advice.

The breastfeeding support programme provided to the intervention group is described below and in Additional file 1. Midwives were instructed to implement the programme, which incorporated the pictures, conversation materials, and an individual breastfeeding plan during antenatal care, and to follow up on the plan during a routine visit 8–12 weeks postpartum. The materials and individual breastfeeding plan were designed to strengthen expectant parents’ self-efficacy regarding breastfeeding, in accordance with the Ten Steps to Successful Breastfeeding and WHO recommendations.

1. Antenatal care:

- Expectant mothers received structured breastfeeding counselling at the antenatal clinic during routine visits at pregnancy weeks 28, 32, and 38 (5–10 min). Week 28: Counselling focused on the health benefits of breastfeeding for both mother and infant. Mothers were provided with an individual breastfeeding plan that included self-study activities. The midwife informed sources of breastfeeding support within the plan and encouraged the expectant mother to complete it together with her partner. The plan included questions about the expectant mother’s intentions, the couple’s experiences and expectations, and the type of breastfeeding support they wanted from each other, their family, and healthcare professionals. Expectant parents were also asked to watch four short online breastfeeding lectures before the visit in pregnancy week 32.
  Week 32: Counselling focused on skin-to-skin contact and initiating breastfeeding immediately after birth. The midwife and mother jointly reviewed the breastfeeding plan, discussing intentions, experiences, expectations, and preferred support. Self-study activities in the breastfeeding plan completed before the visit addressed care routines that facilitate breastfeeding.
  Week 38: Counselling focused on recognising early infant feeding cues and assessing effective breastfeeding.

b. The individual breastfeeding plan included:
1) Self-study activities during pregnancy,
2) QR-codes linking to four short online breastfeeding lectures, and.
3) QR-codes linking to two informational leaflets.

2. Postpartum care
  The midwife followed up on the breastfeeding plan during a routine visit at 8–12 weeks postpartum. Counselling addressed the mothers’ intentions, the parents’ experiences, and the type of breastfeeding support they required from each other, their family, and healthcare professionals. If additional support was needed, mothers were referred to the breastfeeding outpatient clinic.

During the years 2020–2022, partners were not allowed to attend antenatal care visits due to the COVID-19 pandemic. In standard care, approximately 15 min are allocated to discussing breastfeeding during the antenatal visit in pregnancy week 28; however, there are no established guidelines regarding the content of this discussion. Expectant mothers also received a informational paper leaflet during the visit.

### Sample

The 20 expectant mothers who consented to participate were recruited using maximum variation purposive sampling, based on education, age, and parity [25]. The inclusion criteria required that all mothers had lived experience of participating in this breastfeeding programme, were in good health at pregnancy week 24, intended to initiate breastfeeding after birth, and were able to communicate in Swedish.

### Data collection

#### Recruitment process

The midwives (*n* = 20) working in antenatal care within the intervention group were instructed to invite one of the first expectant mothers they encountered during the data collection period to participate. They were advised to ensure diversity in educational background, age, and parity among the selected participants. The expectant mothers were provided with written information about the purpose of the study and the opportunity to participate. Thereafter, in pregnancy week 24, the midwife provided verbal information about the larger research project and this study and invited the mothers to participate. Once the mothers had provided written consent for both the larger research project and this study, baseline data were collected (Table 3). The data on the characteristics of the mothers presented in this study were derived from the baseline data collected as part of the larger research project, completed by the mothers after their visit with the midwife. All participants were contacted by telephone (IB) and asked whether they preferred to take part in an initial telephone interview at a time that suited them or to respond to the same questions via written diary entries submitted by post. The mothers were informed that the interviewer was a female midwife (IB) with experience in providing support to breastfeeding families, as well as in performing qualitative telephone interviews. At two months postpartum, they were again given the choice of either being interviewed or completing a diary. The mothers’ preferred mode of participation, interviews or written diaries during pregnancy and postpartum, is presented in Table 2.

**Table 2:** Mothers who participated in interviews or written diaries (*n* = 20)

Interviews during pregnancy, *n* (%)	10 (50)
Diaries during pregnancy, n (%)	7 (35)
Interviews 2 months postpartum, n (%)	8 (40)
Diaries 2 months postpartum, n (%)	5 (25)

The lived experiences of receiving support from midwives and partners during a structured breastfeeding support programme in antenatal care were explored through written diaries and telephone interviews. Written diaries were chosen to allow mothers sufficient time to reflect on their experiences and to describe both positive and negative aspects of support from midwives and their partners [17]. The research team developed the diary questions based on a literature review and their professional experience. The interviews followed the same structure but offered mothers an opportunity to articulate their unique experiences through dialogue, enabling the interviewer to encourage reflection and facilitate a deeper exploration of their experiences of support from midwives and partners during the structured breastfeeding support programme [17].

The telephone interviews began with a brief explanation of the study’s purpose and confirmation of consent to record the interview. The initial questions in both the diaries and interviews were: *‘Did you visit the midwife in week 28 of pregnancy? Could you please tell me about what the midwife discussed and/or what materials you received regarding breastfeeding at the visit?’* During the interviews, probing questions, such as *‘Can you tell me more?’* or *‘What happened next?’* were used to explore the topic further in line with the study’s aim. The interviews lasted on average 30 min, and partners were not present.

After each interview, field notes were taken to capture contextual details such as the date, time, and atmosphere during the interviews, as well as the interactions between the interviewer and the expectant mother. These notes included the mother’s responses and the interviewer’s own reflections, feelings, and emerging insights or questions arising from the data. This approach aimed at maintaining openness and self-reflection to uncover the mothers’ lived experiences. The notes served as a bridge between the raw data and the interpretative process, thereby deepening the analysis during data interpretation [17].

### Data analysis

The interviews were transcribed verbatim. The written material from both interviews and diaries was read and re-read to gain a sense of the whole, as described by Dahlberg et al. [17]. The researcher (IB) maintained an open attitude and posed reflective questions about the written material, such as: ‘*Do I see anything new in the written material?’* or ‘*Is there something that contradicts what I already know about the material?’* [26]. Statements made by mothers that related to the aim of the study (meanings) were identified and compared [17]. Thereafter, attention shifted towards understanding these meanings, involving comparisons of both similarities and differences. Meanings deemed to be the same were grouped together, resulting in seven themes [17]. This was followed by a search for underlying meanings [17]. Each theme concluded with a synthesis, offering suggestions about why certain conditions or factors facilitate support, whereas others may hinder it during a structured breastfeeding support programme. In the final interpretive step, the research team formulated a main understanding and tested it against the written material. This main understanding suggests a way to comprehend and explain the meaning of the phenomenon as a new, integrated whole.

Throughout the entire interpretation process, the validity of the interpretations was carefully evaluated. The researchers strove to maintain openness by continuously discussing and reflecting on their own expectations and experiences. Different preliminary interpretations of each theme were considered. When these preliminary interpretations were found to be inconsistent with the data or the aim of the study, they were re-examined. The researchers engaged in a continuous movement back and forth between the written material (the original whole), the themes (the parts), and the main understanding (the new whole) [17]. The study follows the Consolidated Criteria for Reporting Qualitative Research for interviews and focus groups [27].

## Results

Characteristics of the participating mothers are shown in Table 3. A total of twenty mothers participated. Their ages ranged from 24 to 36 years; half had a university-level education; half had previous experience of breastfeeding; and the majority were married or cohabiting.

**Table 3:** Characteristics of the mothers (*n* = 20)

Age, mean (range)*	30 (24–36)
University education, n (%)*	9 (50)
Married/cohabiting (%)*	17 (94)
Previous experience of breastfeeding n (%)*	9 (50)

*Data from two mothers are missing

The themes highlight what supports expectant and new mothers in a structured breastfeeding support programme, the conditions required for effective support from midwives and partners, as well as factors that prevent such support. Each thematic analysis ends with suggestions that help explain why certain conditions or factors either favour or prevent support. The findings move from individual themes to a more abstract level, offering an explanation of the phenomenon as a whole. The main interpretation is: Being prepared to make independent decisions about breastfeeding.

### Feeling safe through responsive, individualised dialogue

Expectant and new mothers’ lived experience of support during a structured breastfeeding support programme in antenatal care can be interpreted as a sense of safety when midwives discuss breastfeeding based on the family’s needs. Mothers appreciate midwives who foster open, reflective dialogue. They describe how midwives ask questions; listen to their thoughts, experiences, and concerns; and confirm their understanding. They also appreciate when midwives provide self-study materials and inform them in advance about what will be discussed at the next appointment.

*The midwife asked whether my partner and I had any thoughts about breastfeeding*,* what perceptions I have of breastfeeding based on experiences from my surroundings*,* and what ambitions I have myself. Then I was given My/Our Breastfeeding Plan to read and complete before the next visit… I had the opportunity to ask questions and received tasks for the next time. It was a pleasant conversation.* (Expectant mother 12)

*She took notes about how my experience had been last time*,* how it had gone*,* what support I had received and what I had not received*,* and what I now wished for… what I wanted to be different this time. It felt good.* (Expectant mother 4)

When expectant mothers feel safe with their midwives, they are more likely to ask questions and share their thoughts and experiences. This is particularly crucial for mothers expecting a subsequent child who have had previous experiences with breastfeeding that have been negative. They feel hopeful that breastfeeding will go better this time and that they will receive better support. Mothers feel encouraged to ask for additional support and to be more assertive at the child healthcare centre. They are satisfied when midwives document their experiences, wishes, and support needs in their medical journals. However, some mothers report that these dialogues can revive past negative feelings, worries, and fears of failure. Overall, support from midwives boosts their confidence to attempt and persevere with breastfeeding.

*I think it feels good; I have a good relationship with her… I feel safe and can say exactly what I think and feel. I don’t feel that there’s anything I need to hide; I can be honest*. (Expectant mother 4)

*During the pregnancy*,* I have felt a bit uncertain and afraid that it would not work and that I would fail… But… I now feel stronger in my decision to try to make it work.* (Expectant mother 10)

*I’m really happy that I succeeded this time*,* and I had a lot of support—from my midwife*,* whom I trusted greatly. It somehow became less dramatic. Before*,* it felt more like a compulsion*,* and as though it was taboo not to breastfeed. When it goes in all directions—shame or guilt or… it just becomes more stressful*,* as if you have to make it work. This time*,* there was none of that; nothing like that was expressed by anyone. The focus was only on the positive. Like*,* it’s something good and that support is available. If it doesn’t work*,* then it doesn’t work. And for me*,* that has been really good *(Mother 10, 2 months after birth).

In summary, within the structured breastfeeding support programme, support emerges as a relational phenomenon in which a sense of safety is created through responsive and individualised dialogue that acknowledges mothers’ lived experiences and life situations. Through this dialogical process, vulnerability and uncertainty are held and transformed into trust and a lived sense of possibility, enabling mothers to engage with breastfeeding as meaningful and manageable rather than imposed.

### Grounding confidence through tailored and accessible breastfeeding knowledge

Expectant mothers find support from midwives and partners during a structured breastfeeding support programme beneficial for gaining knowledge through videos and leaflets. Those with little or no prior knowledge learn about positioning, latching the infant to the breast, expressing milk, and the normality of frequent feeding. They value learning that initial challenges, such as sore nipples, pain, and low milk supply, are common, and that the breastfeeding clinic can offer support. Others appreciate revisiting familiar information and focusing on addressing previous challenges. After giving birth, new mothers feel reassured knowing that frequent feeding, including during the night, is normal.

*She has been waking up about every other hour. I haven’t really been worried about it. Instead*,* it has actually been a relief; I’ve thought – well*,* that’s good*,* she’s making sure everything is working*,* sort of. Rather than thinking it’s annoying to wake up all the time.* (Mother 4, 2 months after birth)

How information is provided is important. Some expectant mothers learn best by listening to and watching videos of other breastfeeding mothers. Those who find it difficult to absorb large amounts of information at once prefer to receive it in smaller portions. Mothers feel supported when midwives provide practical demonstrations and focus on what is most relevant for the early postpartum days.

*I started reading a bit and thought*,* how and when am I supposed to know all this? We take in the information little by little… The midwife showed me*,* using one of those crocheted breasts… the colostrum… how to express it.* (Expectant mother 15)

In summary, mothers describe support as gaining accessible, well‑timed, and individually adapted breastfeeding knowledge that makes early challenges feel understandable and expected. Through varied formats and practical guidance, information becomes a calming resource that strengthens preparedness and confidence after birth.

### Breastfeeding as an existential anchor for emotional closeness and maternal identity

Expectant mothers anticipate that breastfeeding will make them feel needed and help establish the first and closest emotional relationship with their infants. Those who have already decided to breastfeed do not always wish to discuss it with others; they may have informed their partner of their decision and prefer to read and learn about breastfeeding from trusted sources. New mothers feel empowered knowing that decisions about breastfeeding are made by them and their infants.

*We read in the… leaflet. He said*,* “Well*,* it’s not me who is breastfeeding”… He doesn’t have many opinions; he knows that I found it difficult when it didn’t work out with [name]. It felt like something was taken away from me*,* that I couldn’t continue breastfeeding. That… everyone would rush in and… feed… because I couldn’t breastfeed… Now I’m going to start crying; for me*,* it’s one of my greatest fears – that they would bond with someone else instead of with me.* (Expectant mother 4)

*It is important to read about*,* and be strengthened by*,* the knowledge that it is myself and the baby who make the decisions.* (Mother 1, 2 months after birth)

In summary, support carries an existential dimension, revealing breastfeeding as more than nutrition—it becomes a way to affirm closeness, belonging, and identity as a mother. This meaning is strengthened when autonomy in decision-making is respected and supported.

### Co‑creating space for breastfeeding: partner support as negotiated meaning

Expectant mothers feel that self-study materials encourage communication between parents. They find it supportive to discuss their thoughts, experiences, and expectations about breastfeeding. For example, they talk about managing sleepless nights, how the partner can support breastfeeding, and the need for better support from healthcare professionals.

*We have talked about being more assertive this time in seeking help from healthcare professionals if breastfeeding does not work. That we really want to make it work this time. That we will support each other*,* and that the father will take more responsibility for our other child during breastfeeding.* (Expectant mother 3)

Expectant parents with older children often share past breastfeeding experiences. Partners of older children sometimes report feeling left out and describe bonding as having taken longer. Some expectant parents consider expressing milk as a way to involve the partner. However, partners who wish to bottle-feed immediately after birth are not perceived as supportive when mothers intend to breastfeed exclusively.

In summary, partner support is revealed as a negotiated, relational practice in which dialogue, shared reflection, and practical re‑orientation create space for the mother–infant intention to breastfeed. Involvement is experienced as truly supportive when it aligns with and safeguards this intention; actions that diverge from it (e.g. early bottle‑feeding) may be perceived as undermining rather than helpful.

### Weighing breastfeeding as a lived balance of perceived gains and everyday constraints

Expectant mothers weigh the pros and cons of breastfeeding during a structured programme. They perceive that the materials provide knowledge about specific benefits, including health advantages, the prevention of constipation, bonding, financial savings, and convenience. One mother describes:

*When we watched those films*,* we talked about how good it was to exclusively breastfeed… the breast milk*,* how beneficial it was… and that it was good for me too… So I felt that… everyone benefits from it. And that my partner… he would take responsibility for changing nappies and getting up more during the nights… At first*,* we had said that we would combination feed*,* because he wanted to be involved in feeding. But then we decided that I would exclusively breastfeed instead*. (Expectant mother 15)

Expectant mothers also recognise some disadvantages. They worry that their partner might feel excluded, unable to comfort the infant, or unable to bond closely. Some fear that their partners might not find them sexually attractive while breastfeeding. Other concerns include the perception that breastfeeding restricts individual time and activities, such as exercising. Talking to family and friends about breastfeeding and formula feeding is experienced as supportive; however, friends and family often hold positive attitudes towards formula feeding, suggesting that infants sleep better at night when bottle-fed.

In summary, breastfeeding is experienced as a lived tension between anticipated gains (health, bonding, financial savings, convenience) and relational, bodily and temporal concerns (partner inclusion, intimacy, autonomy, time). Support emerges when this weighing is held within respectful dialogue, enabling decisions that feel coherent, sustainable, and compatible with everyday family life.

### Trust as a foundation for perseverance and acceptance

New mothers trust their midwife’s advice on breastfeeding and the materials provided during visits. This trust encourages them to persevere with breastfeeding despite difficulties, as they want their infant to receive breast milk, even if only in small amounts each day. However, knowing the benefits of breastfeeding can also cause sadness when mothers are unable to breastfeed as much as they would like:

*It becomes a cosy moment*,* and he gets… a little breast milk at least… It’s a shame he might have been able to have just a little bit more.* (Mother 19, 2 months after birth)

In summary, trust in midwifery guidance and materials is experienced as a sustaining factor that enables mothers to persevere despite breastfeeding difficulties. This trust carries an existential ambivalence: awareness of the benefits of breastfeeding can deepen commitment while also intensifying feelings of sadness or loss when breastfeeding cannot be realised as hoped, inviting acceptance.

### Feeling left behind when dialogue and continuity are missing

If midwives provide breastfeeding materials without engaging in dialogue, expectant and new mothers feel unsupported and may disregard the materials. This can also happen if their regular midwife is unavailable, and they are seen by another midwife or a student. Other barriers include arriving late to visits, not having time to use self-study materials due to the demands of older children, and feeling lonely or insecure because partners were not allowed to attend visits due to COVID-19 restrictions. These mothers struggle to understand the information they receive about beastfeeding and feel that they still do not know much about it. They may also be unsure of which questions to ask or feel unable to ask them.

Mother: *During pregnancy*,* I didn’t get much information… I got a QR code that I could scan and watch a film*,* she said. But we didn’t really talk… about it.*

Interviewer: *Did you watch the films?*

Mother: *No*,* I didn’t. It’s my second child*,* so I didn’t even know if I wanted to breastfeed in the beginning. No*,* to be honest*,* I didn’t have a very good midwife*,* so she didn’t talk much about anything… I didn’t feel safe with her.* (Mother 16, 2 months after birth)

In summary, the absence of responsive dialogue and relational continuity reveals a breakdown of support: information loses meaning, mothers feel excluded and unprepared, and questions remain unasked or unformulated. Such disruption highlights that support is not merely the distribution of materials but the creation of a relational space for understanding, connection, and readiness.

### Main interpretation

The meanings of expectant and new mothers’ lived experiences of support from midwives and partners during the programme can be understood as being prepared to make independent decisions about breastfeeding. Preparedness is not limited to receiving information; rather, it is lived as a relational process through which breastfeeding becomes meaningful, manageable, and personally owned throughout pregnancy and early motherhood. Support from midwives is primarily experienced as dialogical and relational. When midwives engage in responsive, individualised conversations that acknowledge mothers’ experiences, concerns, and life situations, a sense of safety is established. This sense of safety enables mothers to express uncertainty and ask questions, thereby strengthening confidence and orientation during both pregnancy and the postpartum period. Within such relationships, information is not experienced as instruction to be followed, but as something that can be reflected upon and integrated into the mother’s own understanding. Preparedness is further grounded in accessible and tailored breastfeeding knowledge, provided in appropriate formats and at a pace that corresponds to mothers’ needs.

Breastfeeding is also lived as an existential concern, closely connected to emotional closeness and maternal identity. Being prepared, therefore, involves having these meanings acknowledged and respected by both midwives and partners. Partner support emerges as a negotiated relational practice in which shared reflection and practical alignment help to safeguard the mother–infant intention to breastfeed.

Trust in midwifery guidance functions as a sustaining foundation that supports perseverance and acceptance when breastfeeding is challenging. Conversely, when dialogue or relational continuity is lacking, support loses its meaning and mothers experience themselves as unprepared. Preparedness thus arises not from information alone, but from being held within a responsive relational space that fosters understanding, confidence, and autonomy.

## Discussion

This study explored the meanings of expectant and new mothers’ lived experiences of support from midwives and partners during a structured breastfeeding support programme in antenatal care and revealed seven themes: Feeling safe through responsive, individualised dialogue; Grounding confidence through tailored and accessible breastfeeding knowledge; Breastfeeding as an existential anchor for emotional closeness and maternal identity; Co‑creating space for breastfeeding with partner support as negotiated meaning; Weighing breastfeeding as a lived balance of perceived gains and everyday constraints; Trust as a foundation for perseverance and acceptance; and Feeling left behind when dialogue and continuity are missing. The main interpretation can be understood as being prepared to make independent decisions about breastfeeding.

Our study demonstrates that the manner in which expectant mothers were supported through a structured breastfeeding support programme was based on a trustful, reflective dialogue between them and their midwives. The materials used by the midwives encouraged them to listen, ask open-ended questions, reflect, and summarise the responses [18]. The way in which midwives communicate with mothers about breastfeeding is therefore crucial. They should use this dialogue to tailor the support to the mother’s individual wishes and needs. This approach can be understood as providing support based on the principles of the philosopher Kierkegaard. He asserts that, in order to truly help someone, the helper must first understand the person —both physically and mentally—and begin from that point. This requires humility and patience. He emphasises that genuine help is about serving rather than dominating, which requires setting aside one’s own pride and acknowledging the possibility of being wrong [28]. Expectant and new mothers preferred to discuss breastfeeding with a midwife they had met on several occasions. This indicates that continuity is important for enabling a mutual, trusting dialogue about breastfeeding. In Sweden, midwives working in antenatal care often establish long-term relationships with their patients. They typically meet expectant mothers eight to ten times during pregnancy and follow up on new mothers after delivery [19]. This setup facilitates a trusting dialogue about breastfeeding.

A systematic review has shown that antenatal breastfeeding education can improve breastfeeding self-efficacy among mothers with previous unsuccessful breastfeeding experiences [8]. Our study describes these mothers’ feelings of resentment, as such experiences may revive past negative feelings, worries, and fears of failure. Therefore, some expectant mothers require additional support [29, 30].

The expectant and new mothers felt that the breastfeeding programmes were beneficial for gaining knowledge about how breastfeeding works. Those with little or no prior knowledge valued learning about positioning, how to attach the infant to the breast, and the normality of frequent feeding. They also appreciated understanding that initial challenges are common and that they can receive support from the breastfeeding clinic. Such knowledge is important, as many mothers experience early breastfeeding problems, which is a common reason for stopping breastfeeding earlier than planned [31]. However, this study found that some mothers struggled to absorb large amounts of information and felt more supported when midwives tailored the information to their individual needs. Practical breastfeeding support was perceived as particularly important. Such knowledge is important to convey to healthcare professionals working in maternity and child healthcare services. In fact, data from Europe indicate that nearly half of the population find it difficult to understand, evaluate, and apply health information [32]. Health literacy is suggested to be essential for individuals to make informed decisions in everyday life, to feel good, and to feel in control of their health. Health literacy may vary depending on a person’s knowledge, emotional state, and cultural background. Individuals with lower levels of education or social status often have reduced health literacy [33]. This could be one reason why mothers with lower education levels find antenatal education in small groups less helpful and participate to a lesser extent [13]. More knowledge is needed in this area, particularly relating to breastfeeding, as these mothers also tend to breastfeed for shorter periods [4].

The new mothers felt empowered because the self-study materials strengthened their confidence that breastfeeding decisions were made by the mother and the infant. Expectant mothers felt supported when their partners understood what breastfeeding meant to them and allowed them to make decisions about breastfeeding. Some mothers expressed that breastfeeding allowed them to get to know their infants and create emotional bonds in a peaceful and quiet environment. A previous study indicates that mothers who were forced to wean earlier than they had wished experienced feelings of sadness [34]. Congruent with Palmer, we found that this sadness lingers even when mothers are expecting their next child [30]. A study from Sweden, Australia, and Ireland found that some mothers with a history of bottle feeding perceived the emotional bond with their infant as particularly strong, which motivated them to continue breastfeeding [35]. This indicates that we need more knowledge about mothers’ experiences of motherhood in relation to breastfeeding. Lastly, this study found that expectant mothers who had already decided to breastfeed did not always wish to discuss this decision with others. These mothers may have a strong family history of breastfeeding around them [35].

The expectant mothers felt that the materials encouraged communication with their partners. Co-parenting is seen as a way for parents to start working together [36, 37]. Mothers find it supportive when both parents discuss their thoughts, experiences, and expectations about breastfeeding. Partners with older children shared feelings of being left out and reported that it had taken a long time to bond with the child. This is consistent with a previous study indicating that most infants have a primary person to whom they are attached, even though they often have bonds with other individuals, such as the other parent and siblings [38]. Breastfeeding mothers may experience jealousy from their partners due to the close bond created through breastfeeding between mother and infant [39]. This study shows that when a partner wants to be involved by feeding the infant, this may be seen as unsupportive if it conflicts with the mother’s desire to breastfeed exclusively. This highlights the need for midwives working in antenatal care to involve both parents in a trustful dialogue by listening attentively, using open-ended questions, reflecting, and summarising the parents’ responses. It is also important to find ways to involve partners and help them in establishing their own relationship with the infant, for example, through skin-to-skin contact [19].

Expectant mothers recognised both the pros and cons of breastfeeding. They gained knowledge about its specific benefits, which motivated them to take ownership of their breastfeeding choices, especially when they found ways to involve their partners without compromising the act of feeding the infant. Antenatal educational programmes that provide information about the health benefits of breastfeeding for both infant and mother can influence whether mothers choose to initiate breastfeeding [8].

Our findings indicate that if expectant mothers fear their partners might feel excluded or perceive them as being less sexually attractive during breastfeeding, they may feel unsafe and hesitate to assert their breastfeeding wishes. This aligns with a previous study showing that partners’ attitudes can influence mothers’ decisions to initiate breastfeeding [40]. A Swedish study also found that partners tend to have positive attitudes towards shared feeding, which are influenced by parenting books portraying commercial milk formula as almost equivalent to breastfeeding [19]. Expectant parents may also be influenced by materials aimed at promoting infant health by strengthening the parents’ relationship and cooperation around the infant, which are used in small-group antenatal educational programmes in several regions of Sweden [41]. This material equates bottle feeding and breastfeeding in order to facilitate the partner’s close relationship with the infant and highlights the potential difficulties breastfeeding may pose for the mother [41]. This aligns with blogs and media in high-income countries, where breastfeeding is often framed as anti-feminist [42]. Such messages from midwives conflict with the Ten Steps, which encourage midwives to discuss the importance and management of breastfeeding with expectant and new parents [6]. As a result, parents may perceive breastfeeding as unimportant or become confused by contradictory messages. It is therefore important to recognise that midwives have a significant influence on families’ health-related decisions regarding breastfeeding or formula feeding, due to their professional knowledge and the high level of public trust in them [42].

### Implications for research and practice

Our findings can guide midwives in tailoring support to expectant and new mothers, enabling mothers to experience the support as meaningful and positive. It is important that antenatal educational programmes are offered in both small group settings and through individual face-to-face sessions in order to reach vulnerable groups, such as parents with low levels of education [13], who tend to breastfeed for shorter durations [4]. While self-study materials on breastfeeding can be a useful complement, they cannot replace the interaction between a midwife and the mother. Such dialogue is central to providing support and enables care to be tailored to the family’s specific wishes and needs.

### Methodological considerations

A lifeworld hermeneutic approach [17] was used to analyse the results, as it was deemed appropriate for capturing the lived experiences of expectant and new mothers. This approach contributed to the study’s validity by aligning with the quality criterion of describing participants’ lived experiences. To gain insight into experiences over time, data were collected through diary entries and semi-structured individual interviews conducted during pregnancy and at two months postpartum. In lifeworld research, the interviewer seeks rich and detailed descriptions; however, participants may at times provide responses they believe the researcher expects [17]. In this study, follow-up interviews were conducted by the same interviewer, who had professional experience in the field. This continuity may have helped mothers feel more comfortable, making it easier for them to reflect openly and share honest accounts of the research phenomenon. The longitudinal design further allowed for a comprehensive understanding of how mothers’ experiences evolved over time. The findings included translated quotations that reflected the mothers’ lived experiences. Objectivity, a key quality criterion in lifeworld research, was prioritised. The researchers sought to maintain an open attitude towards the phenomenon by critically examining the data and continuously reflecting on interpretations throughout the research process. Additionally, the research team engaged in both individual and collaborative reflections during the study [17, 26]. Generalisation, another essential quality criterion in lifeworld research [17], involves interpreting findings within their contextual framework. In this study, all expectant mothers were recruited from a single region in Sweden and had expressed an intention to breastfeed when asked at 24 weeks of pregnancy. Most participants were married or cohabiting, with an average age of 30 years. A more diverse sample might have provided alternative perspectives on experiences of support during a structured breastfeeding support programme.

## Conclusions

Support within a structured antenatal breastfeeding programme can be understood as being prepared to make independent decisions about breastfeeding. Preparedness is not limited to receiving information; rather, it is lived through everyday encounters in which support is experienced as meaningful and relevant. Preparedness develops when midwives engage in trustful and responsive dialogue, listen attentively to expectant mothers’ experiences, and adapt discussions to individual needs and life situations. This creates a sense of safety that enables mothers to ask questions, reflect, and take ownership of their breastfeeding decisions during pregnancy and after birth. Trust in midwifery guidance further supports perseverance when breastfeeding proves challenging.

Being prepared is also shaped in relation to partners. Mothers feel supported when partners understand what breastfeeding means to them and respect their wishes, allowing shared decision‑making without undermining maternal autonomy. When dialogue and continuity are missing, support loses meaning and mothers may feel unprepared.

## Supplementary Information

Below is the link to the electronic supplementary material.

Supplementary Material 1

## Data Availability

The datasets used and/or analysed during the current study are available from the corresponding author upon reasonable request.

## Abbreviations

BFHI: Baby-friendly Hospital Initiative
Ten Steps: Ten Steps to Successful Breastfeeding
UNICEF: United Nations Convention on the Rights of the Child
WHO: The World Health Organisation
